# Treatment of Hyperammonemia by Transplanting a Symbiotic Pair of Intestinal Microbes

**DOI:** 10.3389/fcimb.2021.696044

**Published:** 2022-01-05

**Authors:** Jing Liu, Chongkai Zhai, Jung-Rae Rho, Sangbum Lee, Ho Jin Heo, Sangwoo Kim, Hyeon Jin Kim, Seong-Tshool Hong

**Affiliations:** ^1^ Department of Biomedical Sciences and Institute for Medical Science, Chonbuk National University Medical School, Jeonju, South Korea; ^2^ Department of Oceanography, Kunsan National University, Kunsan, South Korea; ^3^ Division of Applied Life Science [Brain Korea (BK) 21 Plus], Institute of Agriculture and Life Science, Gyeongsang National University, Jinju, South Korea; ^4^ JINIS BDRD Institute, JINIS Biopharmaceuticals Inc., Wanju, South Korea; ^5^ SNJ Pharma Inc., BioLabs Los Angeles (LA) in the Lundquist Institute for Biomedical Innovation at Harbor-University of California, Los Angeles (UCLA) Medical Center, Torrance, CA, United States

**Keywords:** hyperammonemia, intestinal microbe, ammonia, neurotoxic, pharmabiotic

## Abstract

Hyperammonemia is a deleterious and inevitable consequence of liver failure. However, no adequate therapeutic agent is available for hyperammonemia. Although recent studies showed that the pharmabiotic approach could be a therapeutic option for hyperammonemia, its development is clogged with poor identification of etiological microbes and low transplantation efficiency of candidate microbes. In this study, we developed a pharmabiotic treatment for hyperammonemia that employs a symbiotic pair of intestinal microbes that are both able to remove ammonia from the surrounding environment. By a radioactive tracing experiment in mice, we elucidated how the removal of ammonia by probiotics in the intestinal lumen leads to lower blood ammonia levels. After determination of the therapeutic mechanism, ammonia-removing probiotic strains were identified by high-throughput screening of gut microbes. The symbiotic partners of ammonia-removing probiotic strains were identified by screening intestinal microbes of a human gut, and the pairs were administrated to hyperammonemic mice to evaluate therapeutic efficacy. Blood ammonia was in a chemical equilibrium relationship with intestinal ammonia. *Lactobacillus reuteri* JBD400 removed intestinal ammonia to shift the chemical equilibrium to lower the blood ammonia level. *L. reuteri* JBD400 was successfully transplanted with a symbiotic partner, *Streptococcus rubneri* JBD420, improving transplantation efficiency 2.3×10^3^ times more compared to the sole transplantation while lowering blood ammonia levels significantly. This work provides new pharmabiotics for the treatment of hyperammonemia as well as explains its therapeutic mechanism. Also, this approach provides a concept of symbiotic pairs approach in the emerging field of pharmabiotics.

## Introduction

Ammonia is produced as a byproduct of amino acid catabolism in the body. The kidney also produces ammonia from glutamine in the proximal tubule, which is released into the blood circulation system or excreted after concentration in the medullary interstitium (Weiner et al., 2015; Liu et al., 2018). When the glucose level is decreased by starvation or intense exercise, the skeletal muscle and other peripheral tissues also generate a significant amount of ammonia through amino acid catabolism (Kamei et al., 2020). Ammonia is a highly neurotoxic compound at even sub-millimolar concentrations (Jin et al., 2018). The strong toxicity of ammonia makes the detoxification pathways of ammonia indispensable in animals. Ammonia in mammals is rapidly converted to a nontoxic nitrogenous compound, urea, in the liver through the urea cycle for its eventual excretion in the urine (Jover-Cobos et al., 2014; Meng and Wang, 2018).

When the urea cycle does not function properly by either an inherited genetic disease or liver damages, the blood ammonia level elevates to cause hyperammonemia (Liu et al., 2018; Soria and Brunetti-Pierri, 2019). The elevated ammonia seriously damages the brain, leading to severe consequences in the central nervous system (CNS) (Oja et al., 2017;, Butterworth, 2020). The consequences of the CNS damaging by ammonia typically are represented as alteration on mood and personality, cognitive impairment, ataxia, convulsions, and coma (Karanfilian et al., 2020). Considering such strong neurotoxicity of ammonia even at a sub-millimolar concentration, it would be reasonable to suspect ammonia as a possible etiological agent for Alzheimer’s Disease (Jin et al., 2018).

Because of the high incidence rate of hyperammonemia resulting from liver diseases, kidney diseases, and genetic defects in the urea cycle, therapeutic approaches to treat hyperammonemia are being actively investigated (Huh and Farrell, 2011; Machado and da Silva, 2014; Ghallab et al., 2016; Li et al., 2019; Matsumoto et al., 2019). One target of the current therapeutic methods is the reduction of ammoniagenesis and its absorption in the gastrointestinal (GI) tract by using lactulose, sodium benzoate, and rifaximin. Another is the activation of ammonia removal by upregulating ureagenesis either through treatment with N-carbamylglutamate or through supplementation of urea cycle intermediates and synthesis of glutamine using sodium phenylacetate, sodium phenylbutyrate, l-arginine, l-citrulline, and carglumic acid (Wright et al., 2011; Liu et al., 2018). However, these medications do not provide a satisfactory result in terms of efficacy. Also, these medications have been shown to cause serious adverse effects, including abdominal cramping, flatulence, bloating, electrolyte imbalance, nausea, diarrhea, and acute GI bleeding (Liu et al., 2018). Therefore, pursuits to an efficient treatment for hyperammonemia remain active.

Recently, an increasing amount of evidence has shown the critical roles of metabolisms in the gut for maintaining human health (Chen et al., 2013). The intestine is the site where the digested nutrients are absorbed through intestinal capillaries and lymphatic vessels (Sommer and Bäckhed, 2013; Lee and Hase, 2014; Chung et al., 2018). However, some molecules such as ammonia do not flow unidirectionally. The intestine secretes and absorbs ammonia through passive diffusion and specific transporters such as RhBG and RhCG (Handlogten et al., 2005; Gruswitz et al., 2010). Based on the bidirectional movement of ammonia in the gut, it is theoretically possible that the removal of ammonia in the intestine could facilitate its transportation from blood to the intestine, lowering blood ammonia concentration through a chemical equilibrium (Rahimi and Rockey, 2016). In this regard, the administration of *Lactobacillus* strains showed some degree of efficacy for hyperammonemia in animal experiments although its therapeutic mechanisms have not been elucidated (Nicaise et al., 2008; Ott and Vilstrup, 2014; Shen et al., 2015; Singh et al., 2018; Saeedi et al., 2020).

In this work, after elucidating the therapeutic mechanism of probiotic approaches for lowering blood ammonia levels, we developed a pharmabiotic treatment for hyperammonemia that employs symbiotically-related intestinal microbes that remove ammonia from its surrounding environment. This work not only provides a new approach for effective treatment of hyperammonemia but also explains why probiotics are effective in lowering blood ammonia levels. More importantly, this approach takes the advantage of the symbiotic interactions between gut microbes rather than using genetic engineering of microbial genes, providing safer clinical applications in the future.

## Results

### Blood Ammonia Is in an Equilibrium Relationship With Intestinal Ammonia

**Figure 1:**
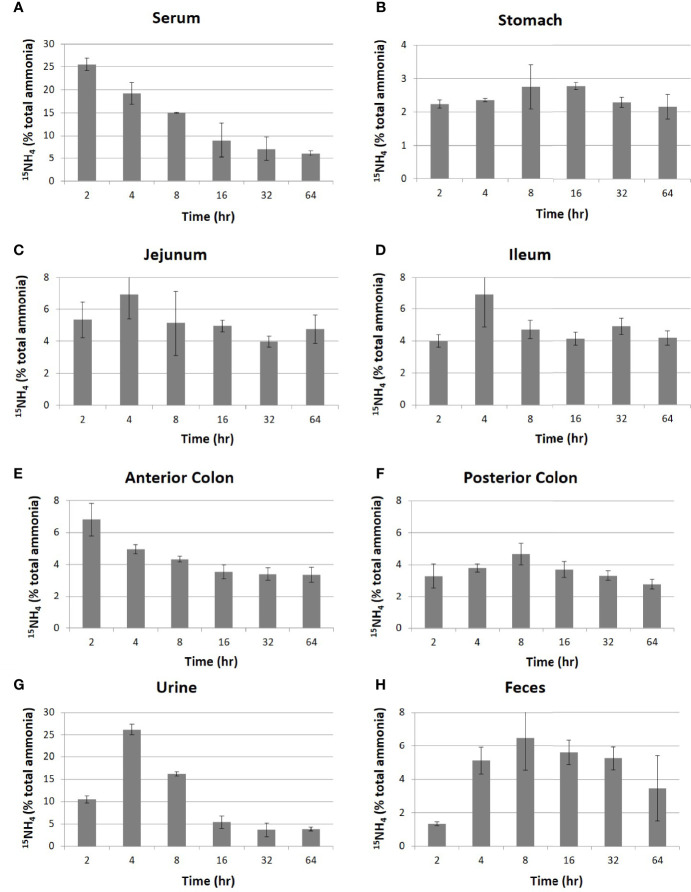
Distribution of intravenously injected ^15^NH_4_ in mouse organs. After intravenous injection of ^15^NH_4_Cl into 4-week-old C57BL/6 mice (n=5 per group) at a dose of 250 mg/kg of body weight, samples of blood, contents of the intestine (jejunum and ileum), contents of the colon (anterior colon and posterior colon), urine, and feces were collected at the indicated time. The migration of ^15^NH was traced by analyzing with high-resolution LC-MS/MS in serum **(A)**, stomach **(B)**, jejunum **(C)**, ileum **(D)**, anterior colon **(E)**, posterior colon **(F)**, urine **(G)**, and feces **(H)**. The quantities in Y-axis were represented in a relative quantity of ^15^NH_4_ in total ammonia. All data were expressed as the mean ± SD, as indicated.

Nonpolar small molecules such as ammonia could potentially be in equilibrium between intestinal lumen and blood. Blood ammonia generated within tissues would enter the intestinal lumen. On the same principle, intestinal ammonia generated by bacterial fermentation should move to blood. Considering ammonia’s nonpolar nature and its small molecular weight, we first tested the equilibrium relationship between blood ammonia and intestinal ammonia by intravenously injecting the radiolabeled ammonia, ^15^NH_4_Cl, to mice. The concentrations of ammonia (^14^NH_4_Cl, **Figure S1**) as well as radiolabeled ammonia (^15^NH_4_Cl, **Figure 1**) in the blood, urine, feces, intestine (jejunum and ileum), and colon (anterior and posterior) were measured using an ultrahigh-resolution LC/MS/MS to trace to where the body ammonia travels. It was observed that the blood concentration of the injected ^15^N-ammonia rapidly dropped (**Figure 1A**) as the administered ammonia was diffused to other parts of organs, converted into urea, and released into the urine (**Figures 1B–H**). Unlike ^15^NH_4_, the concentrations of ^14^NH_4_ did not change significantly in each organ as expected because ^14^N is the main element of nitrogen (**Figure S1**). The concentrations of ^15^N-ammonia of the intestinal lumen from the jejunum to the anterior colon reached the plateau of 4~6% after small fluctuation within 64 hours after the injection of ammonia unlike that in the stomach (**Figures 1B**–**E**). This suggests that blood ammonia is in an equilibrium relationship with intestinal ammonia. The unabsorbed ammonia in the colon was shown to be released through feces (**Figure 1H**). Since the blood ammonia is in equilibrium with intestinal ammonia from the jejunum to the anterior colon, removal of intestinal ammonia would result in a drop of blood ammonia by shifting the chemical equilibrium.

### The Blood Ammonia-Lowering Lactobacilli Strains Which Remove Ammonia From Their Surrounding Environments Were Identified

**Figure 2:**
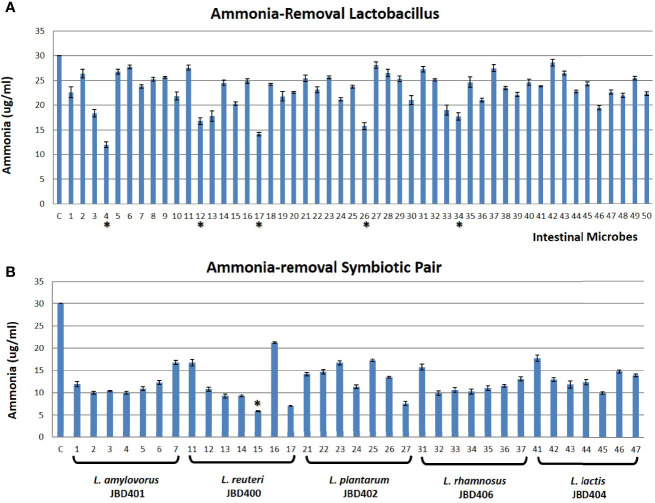
*In vitro* identification of a symbiotic pair of ammonia removal microbes. **(A)** The *in vitro* screening result of *Lactobacillus* strains (Gut Microbiota Bank) having an ability to absorb ammonia from its surrounding environment. X-axis showed untreated control (C) and 50 tested *Lactobacillus* as shown in **Table S1** while Y-axis is the ammonia concentration in the media. The top 5 *Lactobacillus* strains with strong ammonia-removal capacity were indicated as *. **(B)** The representative figure of the ammonia-removing ability of *Lactobacilli* with symbiotic partners (Gut Microbiota Bank) *in vitro*. X-axis indicated untreated control (C) and *L. amylovorus* JBD401 (No. 1~7), *L. reuteri* JBD400 (No. 11~17), *L. plantarum* JBD402 (No. 21~27), *L. rhamnosus* JBD406 (No. 31~37), and *L. lactis* JBD404 (No. 41~47) alone (1, 11, 21, 31, 41) or with symbiotic partner *S. mutans* JBD423 (No. 2, 12, 22, 32, 42), *S. ratti* JBD428 (No. 3, 13, 23, 33, 43), *S. intermedius* JBD429 (No. 4, 14, 24, 34, 44), *S. rubneri* JBD420 (No. 5, 15, 25, 35, 45), *S. lutetiensis* JBD421 (No. 6, 16, 26, 36, 46), or *S. pneumoniae* JBK1-00101 (No. 7, 17, 27, 37, 47) while Y-axis is the ammonia concentration in the media. The symbiotic pair with the best ammonia-removal capacity was indicated as *. All data were expressed as the mean ± SD, as indicated.

Although it has been reported that *Lactobacillus* strains lowered blood ammonia levels efficiently in animal experiments (Singh et al., 2018), the effect of feeding *Lactobacilli* on blood ammonia levels was not known. Since the equilibrium relationship between intestinal ammonia and blood ammonia (**Figure 1**) suggested that the removal of ammonia in the intestine would lower blood ammonia level, we tested the possibility. A total of 50 *Lactobacillus* and *Lactococcus* species were obtained as the candidate strains after the preliminary screening of gut microbes (www.gutmicrobiotabank.com) based on their capability to absorb ammonia in a culture medium (**Table S1**). As shown in **Figure 2A**, most *Lactobacilli* efficiently removed ammonia in the medium by absorption. Among the tested candidate strains, four *Lactobacillus* strains, L4 (*L. amylovorus* JBD401*)*, L12 (*L. reuteri* JBD400), L17 (*L. plantarum* JBD402), L26 (*L. rhamnosus* JBD406), and one *Lactococcus* strain, L34 (*L. lactis* JBD404) displayed an efficient removal of ammonia. Until now, only two *Lactobacillus* species, *L. amylovorus* and *L. plantarum*, were reported to have a therapeutic efficacy on hyperammonemia (Nicaise et al., 2008; Singh et al., 2018). It is worth noting that our screening experiments included all of the previously known *Lactobacilli* with ammonia-lowering capabilities. The experimental results in **Figures 1**, **2A** together with the previous reports indicate that the removal of intestinal ammonia by intestinal *Lactobacilli* could reduce blood ammonia levels through the shift of chemical equilibrium from blood to intestine.

### Co-Culturing Lactobacilli With Their Symbiotic Partner Strains Boosted Their Ammonia-Removing Ability

Based on the nature of microbes, it is likely that most intestinal microbes maintain a symbiotic relationship with others in the gut. Since *Lactobacilli* are one of the main intestinal microbes constituting gut microbiota, we aimed to find symbiotic intestinal microbes for the identified *Lactobacilli* that can further enhance its capability in lowering blood ammonia levels. The cultures of *L. amylovorus* JBD401, *L. reuteri* JBD400, *L. plantarum* JBD402, *L. rhamnosus* JBD406, and *L. lactis* JBD404 were used to find symbiotic intestinal microbes for *Lactobacilli*. From the screening of 697 species (1,378 strains) of the intestinal microbes from Gut Microbiota Bank (https://www.gutmicrobiotabank.com), 6 *Streptococcus* species, *S. mutans* JBD423, *S. ratti* JBD428, *S. intermedius* JBD429, *S. rubneri* JBD420, *S. lutetiensis* JBD421, and *S. pneumoniae* JBK1-00101, were identified to be symbiotically related to the *Lactobacilli* and *Lactococcus* strains. We tested the ammonia-removing ability of *Lactobacilli* or *Lactococcus* strains by co-culturing with the symbiotic *Streptococcus* species (**Figure 2B**).

After considering ammonia-removing abilities as well as the potential for safe human consumption, the co-culture of *L. reuteri* JBD400 and *S. rubneri* JBD420 was further investigated in this study (**Figure S2**). If two species are in a symbiotic relationship, they should prefer being together even in an intestine. We investigated the possibility of a mouse-feeding experiment. As shown in **Table 1**, co-feeding the two strains into mice showed 2.3×10^3^ times more colonization efficiency, confirming the presence of the dramatic symbiotic relationship between the two species in the intestine.

**Table 1:** Comparative analysis of the transplantation efficiency of *L. reuteri* JBD400 with the assistance of the symbiotic partner *S. rubneri* JBD420.

Fed microbes	Counted microbes	Value	Day 0	Day 10	Day 20	Day 40
Single culture of *L. reuteri* JBD400	*L. reuteri* JBD400	Individual value	n.d.	3.2E+03	4.2E+03	4.8E+03
			n.d.	n.d.	n.d.	2.6E+02
			n.d.	n.d.	2.4E+02	9.3E+02
			n.d.	n.d.	n.d.	4.1E+02
			n.d.	7.0E+04	n.d.	n.d.
		average	n.d.	**1.47E+04**	**8.81E+02**	**1.27E+03**
Single culture of *S. rubneri* JBD420	*S. rubneri* JBD420	Individual value	n.d.	2.4E+02	1.1E+03	5.5E+03
			n.d.	n.d.	n.d.	1.4E+02
			n.d.	1.2E+03	n.d.	n.d.
			n.d.	n.d.	9.7E+02	7.7E+03
			n.d.	n.d.	3.3E+03	2.6E+03
		average	n.d.	**2.87E+02**	**1.08E+03**	**3.18E+03**
Symbiotic pair of *L. reuteri* JBD400 & *S. rubneri* JBD420	*L. reuteri* JBD400	Individual value	n.d.	2.2E+04	3.6E+06	8.6E+06
			n.d.	7.5E+05	4.2E+05	9.2E+05
			n.d.	6.3E+02	7.0E+04	3.4E+05
			n.d.	3.1E+03	3.3E+04	4.4E+06
			n.d.	6.5E+05	6.2E+04	2.2E+05
		average	n.d.	**2.86E+05**	**8.40E+05**	**2.91E+06**
	*S. rubneri* JBD420	Individual value	n.d.	1.4E+03	8.9E+04	5.9E+04
			n.d.	7.1E+05	2.2E+05	7.5E+04
			n.d.	3.4E+04	6.8E+04	3.5E+05
			n.d.	6.4E+05	8.1E+03	8.5E+04
			n.d.	7.0E+04	5.3E+04	5.5E+04
		average	n.d.	**2.90E+05**	**8.68E+04**	**1.24E+05**

C57BL/6 mice were daily administered L. reuteri JBD400, S. rubneri JBD420, or symbiotic pair of L. reuteri JBD400 and S. rubneri JBD420, at a dose of 10^9^ colony-forming unit (CFU) for 40 days (n = 5). The fecal samples were collected at the indicated days to quantify the bacterial CFU by serial dilution on MRS for L. reuteri JBD400 or TSA agar medium for S. rubneri JBD420. Results of CFU for the indicated species are expressed as the logarithm of CFU/mL. n.d. indicates bacterial CFU is lower than 100. The bacteria were further identified by 16s rDNA sequencing. Bold Values emphasized the average value from 5 individual values.

### The Symbiotic Pair of the Intestinal Bacteria Treated Hyperammonemia More Efficiently Than the Individual Species Did in Animal Experiments

**Figure 3:**
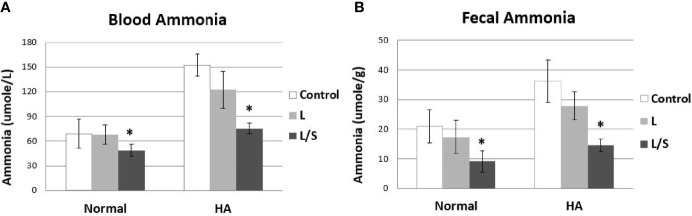
*In vivo* ammonia-removal efficacy of *L. reuteri* JBD400 with the assistance of the symbiotic partner *S. rubneri* JBD420. C57BL/6 mice were daily administered saline (Control), pure culture of *L. reuteri* JBD400 (L), or symbiotic pair of *L. reuteri* JBD400 and *S. rubneri* JBD420 (L/S), as indicated at dose of 10^9^ CFU (n=5 per group) for 2 weeks. After administration of the indicated culture for 2 weeks, each group of mice was injected either saline as control (Normal) or NH_4_Cl at the dose of 100 mg/kg body weight for inducing Hyperammonemia (HA). The ammonia levels in the blood **(A)** and feces **(B)** were collected after 1 hour and quantitated by using a commercial ammonia assay kit. All data were expressed as the mean ± SD, as indicated. The statistical comparisons were analyzed using ANOVA (one-way). All differences were considered statistically significant if p < 0.05. * indicates significant difference compared to the control.

Since the symbiotic pair of *L. reuteri* JBD400 and *S. rubneri* JBD420 showed much higher potential as a therapeutic agent for hyperammonemia in both *in vitro* and *in vivo* colonization experiments, we investigated the therapeutic efficacy of the symbiotic pair for hyperammonemia using a hyperammonemia animal model. The hyperammonemia animal model was made by injecting NH_4_Cl similarly to a previously described method (Singh et al., 2018). In accordance with the *in vitro* and *in vivo* experiments, the symbiotic pair treated hyperammonemia with a much higher efficacy (**Figure 3A**). In normal mice, mice treated with the symbiotic pair of *L. reuteri* JBD400 and *S. rubneri* JBD420 showed a significant reduction in the level of blood ammonia although *L. reuteri* JBD400 caused no changes (**Figure 3A** left). In hyperammonemic mice, the degree of reduction in the level of blood ammonia was much more significant with the symbiotic pair of *L. reuteri* JBD400 and *S. rubneri* JBD420 compared to *L. reuteri* JBD400 treatment (**Figure 3A** right). In agreement with blood ammonia level, fecal ammonia levels of normal mice and hyperammonimia mice were significantly reduced compared to the control group in the treatments of the symbiotic pair of *L. reuteri* JBD400 and *S. rubneri* JBD420, but not in *L. reuteri* JBD400 group (**Figure 3B**). The fecal and blood ammonia levels further validated the experimental results of equilibrium between blood and intestinal ammonia (**Figure 1**).

### The Symbiotic Pair of the Intestinal Bacteria Protected Body Damages by Hyperammonemia in Animal Experiments

**Figure 4:**
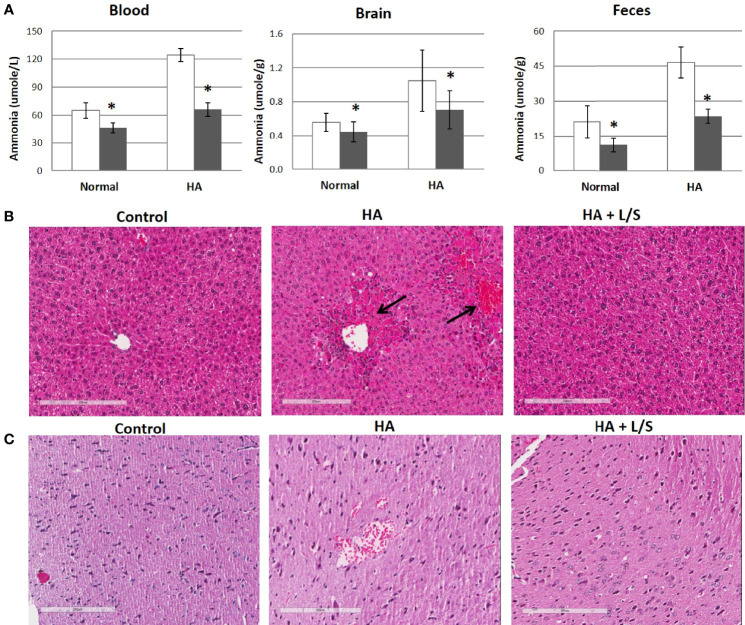
The therapeutic efficacy of the symbiotic pair of *L. reuteri* JBD400 and *S. rubneri* for Hyperammonemia. The symbiotic pair of *L. reuteri* JBD400 and *S. rubneri* JBD420, 10^9^ CFU each in the total volume of 200 μL PBS, was daily administered into each C57BL/6 mice (n = 3 per group) for 40 days. **(A)** Acute hyperammonemia were induced by the injection of TAA at the dose of 250 mg/kg body weight of mice, and ammonia levels were quantitated in the tissue from mice which fed either saline control (open column) or fed symbiotic pair (black column). Normal mice without hyperammonemia induction were analyzed for saline control (open column) or fed symbiotic pair (black column). All data were expressed as the mean ± SD, as indicated. The statistical comparisons were analyzed using ANOVA (one-way). All differences were considered statistically significant if p < 0.05. **(B, C)** After inducing hyperammonemia, the sampled tissues were also analyzed by histological examinations on the H&E-stained section of livers **(B)** and brains **(C)** from control mice without hyperammonemia induction (Control), the hyperammonemic mice (HA), and the hyperammonemic mice fed the symbiotic pair (HA+L/S). Magnifications were × 200. Bar =200 µm. * indicates significant difference compared to the control.

**Figure 5:**
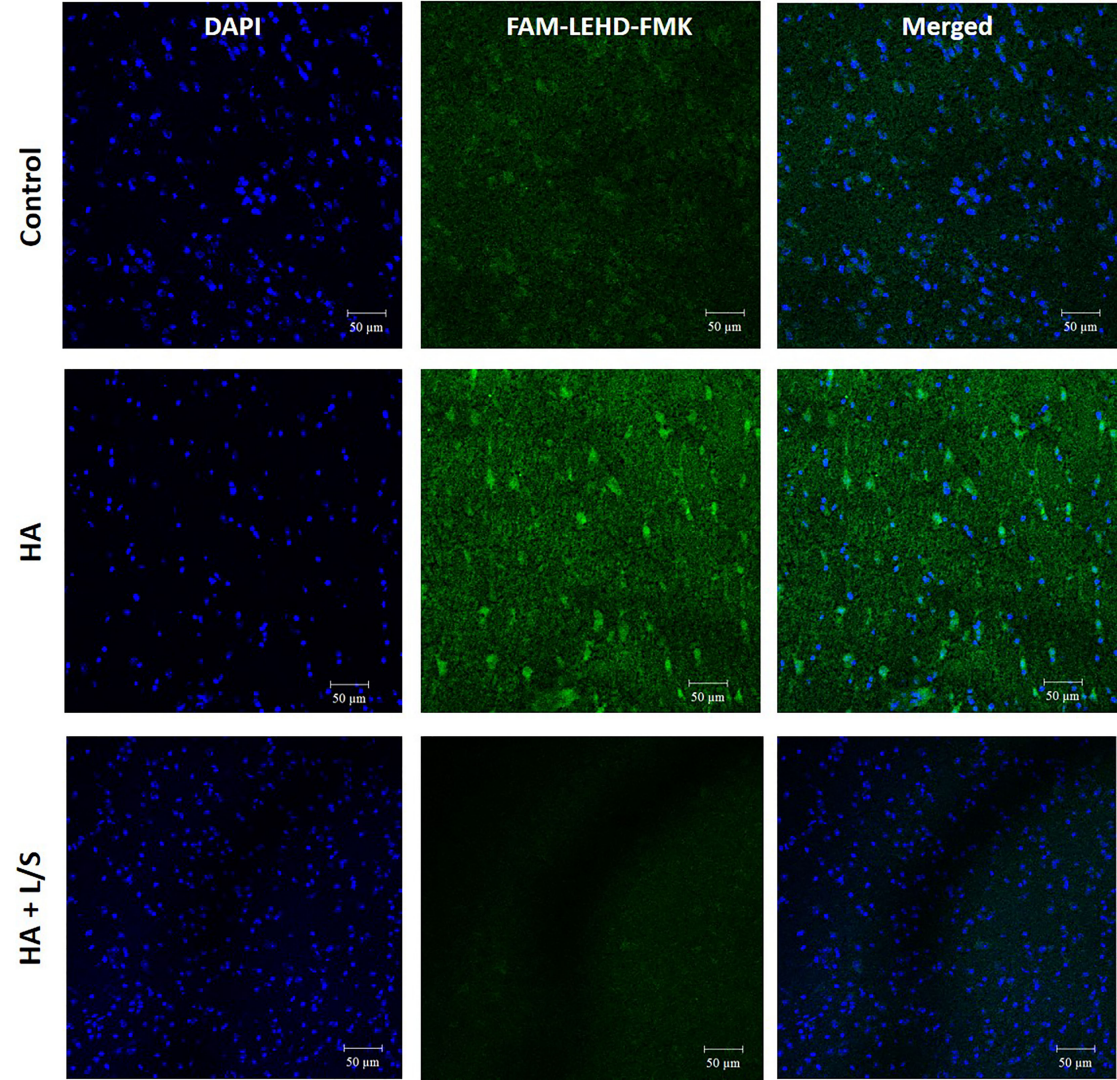
The neuroprotective effect of the symbiotic pair of *L. reuteri* JBD400 and *S. rubneri* in hyperammonemia. The symbiotic pair of *L. reuteri* JBD400 and *S. rubneri* JBD420, 10^9^ CFU each in the total volume of 200 μL PBS, was daily administered into each C57BL/6 mice (n = 3 per group) for 40 days. After inducing Hyperammonemia by injection of TAA at the dose of 250 mg/kg body weight into the mice, histochemical analysis by using the fluorescent inhibitor probe FAM-LEHD-FMK on the brains was performed. HA represents the hyperammonemic mice. HA+L/S represents the hyperammonemic mice fed the symbiotic pair. DAPI represents DAPI staining for nucleus detection and FAM-LEHD-FMK represents the histochemical reaction to detect caspase 9 activation by the fluorescence dye. Magnifications were × 200. Bar =50 µm.

Hyperammonemia provokes body damages, especially in the brain. Following up with the treatment of hyperammonemia by the symbiotic pair, *L. reuteri* JBD400 and *S. rubneri* JBD420, the pair’s effect on hyperammonemia-related body damages was investigated. In our results, a diet constituting the symbiotic pair lowered ammonia levels of the hyperammonemia C57BL/6 mice to normal ranges in blood, brain, and feces (**Figure 4A**). Histological examination on the livers of the above mice group displayed normal lobular architecture with radiating hepatic cords and clear central veins without inflammation or necrosis, which was almost identical to that of the control mice group (**Figure 4B**). In contrast, the thioacetamide-induced acute hyperammonemia mice suffered significant damages to the liver. Severe infiltration of inflammatory cells around the central vein and centrilobular regions, as well as hemorrhage and hepatocyte apoptosis, was observed (**Figure 4B**). Histological examination on the brains of the symbiotic pair group also showed that treatment with the pair prevented neuropathophysiology. Perineuronal vacuolations that indicate brain damages were obvious in the hyperammonemia mice (**Figure 4C**). However, it was obvious that the symbiotic pair prevented brain damages. Labeling of the brain tissues with a fluorescent inhibitor probe FAM-LEHD-FMK showed that the brain damages were caused by the caspase 9-dependent apoptotic pathway (**Figure 5**). The caspase inhibitor-labelling experiment also confirmed that the symbiotic pair prevented ammonia-induced neurotoxicity.

## Discussion

Ammonia is the most common and toxic metabolic waste in the human body. Despite various tissue damages associated with ammonia, neurotoxicity is the most notable complication of hyperammonemia that is clinically represented as encephalopathy (Teperman and Peyregne, 2010) as well as possibly Alzheimer’s disease (Adlimoghaddam et al., 2016; Jin et al., 2018). Both exogenous and endogenous sources are responsible for increased blood ammonia levels (Chen, 2016; Jin et al., 2018). These are some of the endogenous sources: activation of hydrolysis of proteins, degradation of amino acids, deamination of amino-purines, and oxidative deamination of primary amines (Lin et al., 2017; Liu et al., 2018). Malfunction of the urea cycle in the liver (van de Logt et al., 2016; Diez-Fernandez and Häberle, 2017) and excretion of ammonia in kidney are also important players (Nagami and Hamm, 2017; Pourafshar et al., 2018).

Other than endogenous sources of blood ammonia, blood ammonia can be originated exogenously (Lin et al., 2017; Singh et al., 2018). However, the exogenous sources of blood ammonia which originate from bacterial degradation of urea and deamination of amino acids in the GI tract have not received attention because the equilibrium relationship of ammonia between blood and the GI tract was not recognized. In this regard, this work not only contributes to our understanding of mammalian physiology but also provide a new approach to develop a therapeutic agent for hyperammonemia. It should be noted that, however, further investigation is needed to demonstrate the clinical efficacy of the identified microbes due to the limit of induced hyperammonemia using animals in this study.

The equilibrium relationship of ammonia between blood and the GI tract as shown in Fig 1 indicates that ammonia can traverse back and forth between blood and the GI tract, meaning that exogenous sources could contribute a significant amount of ammonia in our body. Furthermore, these results also indicate that the exogenous ammonia generated by intestinal bacteria could lead to hyperammonemia. Although the exogenously supplied ammonia through *i.v.* injection was found in all locations at the GI tract, the peak times of detection were different each other (Fig 1). The peak times were 4 ~ 8 hours after ammonia injection in each location of the GI tract except for the anterior colon (Fig 1E). Interestingly, the peak time and the diminishing pattern of the injected ammonia at anterior colon were same as those of blood (Fig 1A & 1E). This result suggests that anterior colon of the GI tract is the area where traversing of ammonia between body and the GI tract occurs most actively.

In addition, this work could serve to investigate the mystery of the Mediterranean diet which is correlated with a lower risk of Alzheimer’s disease and slower cognitive decline (Estruch et al., 2013; Lourida et al., 2013). Although it is known that the Mediterranean diet is beneficial for brain health, scientific reasoning is unclear. Mediterraneans consume an extraordinary quantity of *Lactobacilli* (Jin et al., 2018; Nagpal et al., 2018). Since ammonia is a highly neurotoxic compound at even a sub-millimolar concentration (Jin et al., 2018), it would be very interesting to investigate the possibility that reduction of the neurotoxic compound, i.e. blood ammonia, by consuming certain *Lactobacilli* in the Mediterranean diet could play a neuroprotective role to lower the risk of Alzheimer’s disease and slower cognitive decline.

The human gut microbiota is a massive and complex microbial community consisting of 100 trillion microbes in the intestine, contributing significantly to human traits as much as our genes, especially in the case of atherosclerosis, hypertension, obesity, diabetes, metabolic syndrome, inflammatory bowel disease, GI tract malignancies, hepatic encephalopathy, allergies, behavior, intelligence, autism, neurological diseases, and psychological diseases (Pandeya et al., 2012; Chen et al., 2013; Chung et al., 2016; Chung et al., 2018). After the realization of the significant role of gut microbiota, pharmabiotic approaches to transplant intestinal bacteria are currently being actively investigated (D’Souza et al., 2012; Chaluvadi et al., 2015; Nguyen et al., 2017). However, sparse transplantation efficiency of candidate microbes is one of the main obstacles in developing efficient pharmabiotics (Nguyen et al., 2017). This work showed that the administration of a symbiotic pair of bacterial strains resulted in a significantly improved transplant efficiency compared to individual administration. This method of transplantation was successful even with strains that have never been fully colonized into the host before (**Table 1**). In this context, we believe that the administration of symbiotic pairs should be actively pursued when developing pharmabiotic medications.

## Materials and Methods

### Materials

Ammonia solution (28%-30%) was purchased from Samchun Pure Chemical CO., Ltd, Korea. Acetonitrile, Urea, Glutamic acid, Ammonium-^15^N Chloride (98% 15N), Urea-^15^N_2_(98% 15N), Camphanic chloride and Ammonia assay kit were purchased from Sigma Chemical Co., St. Louis, Mo, USA. Ethyl alcohol was purchased from Daejung Chemical & Metals CO., Ltd, Korea. The nutrient media used in the research for screening bacteria were Man–Rogosa–Sharpe (MRS, Becton Dickinson, NJ, USA) and Tryptic soy broth (TSB) (Difco, Franklin Lake, NJ).

### Bacterial Strains, Media and Growth Conditions

The bacterial strains used in this study were bought from the Korean Collection for Type Cultures (KCTC), Korean Agricultural Culture Collection (KACC). *Lactobacillus* and *Lactococcus* strains were grown in De Mann Rogosa Sharpe (MRS) broth (Difco™) and *Streptococcus* strains were grown in tryptic soy broth (TSB) (Difco, Franklin Lake, NJ) medium at 37°C anaerobically unless otherwise indicated. *Lactobacillus* and *Lactococcus* strains maintained on MRS agar plates while *Streptococcus* strains maintained on TSB agar plates with routine subculturing at a regular interval of 15 days. After the first screening of *Lactobacillus*, *Lactococcus*, and *Streptococcus*, the high ammonia assimilation ability strains were selected and different probiotics strains were co-cultured in MRS to select the co-culture with the ammonia assimilation ability.

### Animals

All animal care and use protocols were performed strictly in accordance with the ethical guidelines of the Ethics Committee of the Chonbuk National University Laboratory Animal Center, and the animal study protocol was approved by the institution (Approved Number: CBNU 2012-0040) in accordance with the “Guide for the Care and Use of Laboratory Animals,” published by the National Research Council and endorsed by the NIH Office of Laboratory Animal Welfare. Specific-pathogen-free four-week-old female C57BL/6 mice were used in the study. Mice were housed individually in wire-mesh cages in an animal room at a controlled temperature (20 ± 2°C), with a relative humidity of 50%–55%, and exposed to a 12:12 h light/dark cycle. Animals had ad libitum access to standard laboratory rodent chow and fresh sterile water. Five or three mice were included in each group for animal studies as indicated.

### Tracing of Ammonia Distribution in Mice Using ^15^NH_4_Cl

For tracing of radioactivity, ^15^NH_4_Cl at the dosage of 250 mg/kg of body weight was intravenously injected into the tail vein of the mice (n=5 per group). Mice blood, urine, feces, intestine (jejunum and ileum), and colon (anterior colon and posterior colon) were collected at 2 h, 4 h, 8 h, 16 h, 32 h, and 64 h after injection of ^15^NH_4_Cl. The collected samples in an amount of 200 mg or 200 µL were mixed with 400 µL ammonia-free water. After overnight incubation at 4°C, the tubes were vortexed vigorously for 2 min at 13,000 rpm and followed by sonication for 10 min. The mixture was centrifuged for 30 min at 4°C and the supernatants were transferred to new tubes. According to the manufacturer’s protocol, the concentration of ammonia can be quantified by checking the change of glutamate acid. For quantitation of ammonia, 100 µL of assay sample was added to 1 mL ammonia assay reagent, containing 3.4 mM α-ketoglutaric acid and 0.23 mM NADPH. After mixing, it was incubated for 5 min at 18-35°C. The blank glutamate acid samples were obtained. It was mixed with 10 µL of L-Glutamate Dehydrogenase solution and incubated for 5 min at 18-35°C. The total glutamate acid samples were obtained. The glutamate concentration was detected by LC-MS/MS as described previously (Eckstein et al., 2008). The ammonia concentration of the sample was calculated according to the kit protocol.

### *In Vitro* Screening of the Ammonia-Removal Lactobacilli

The fresh culture was prepared from the stock of each *Lactobacillus*, *Streptococcus*, and *Lactococcus* strain and was incubated anaerobically at 37°C for 12 h. Cultures with OD_600_ 0.2-1.0 were obtained at their log/stationary phase. One mL of each culture was transferred into a tube and absorbance was measured at 600 nm using a biophotometer spectrophotometer (Eppendorf). After the addition of 5 µL of ammonium hydroxide (30 µg/mL) to each tube, the cultures were incubated anaerobically at 37°C for 50 min and then centrifuged at 7,000 rpm for 5 min at 4°C. After centrifugation, the supernatants were transferred to a new tube and the ammonia concentrations were measured by an ammonia assay kit (AA0100; Sigma-Aldrich) following the manufacturer’s protocol. The strains with low ammonia quantitation results were selected as ammonia removal microbes.

### Colonization Efficiency of the Symbiotic Pair of the Intestinal Microbes

The intestinal microbe for the colonization test was cultured with appropriate media, washed and resuspended in phosphate-buffered saline (PBS) for mouse feeding. The prepared microbes were orally administered to the mice, 1×10^9^ CFU microbes in 200 µL PBS per day for 40 days (n=5 per group). Assessment of overall health was done including any fatal signs, hair ruffling, change in body weight, fluid consumption, diarrhea, and rectal prolapse. Fecal samples for quantitative bacteriological analysis were collected on day 0 and day 2 after the last administration. A total of 100 mg of feces was suspended in 1 mL PBS and cultured at 37 ± 2°C, for 24-72 h on appropriate, MRS or KF Streptococcus, agar plates. After the gram-straining test, the single colonies were analyzed by PCR and sequencing. The primers for PCR were Lac-spec-F: (5’-GCGGAATTTAAGCA GCGATAC-3’), Lac-spec-R: (5’-CCTGGAAAGCATTAAATCAGG-3’) and Strep-spec-F: (5’-TCGATGCAGAAACAATGACATTGC-3’), and Strep-spec-R: (5’-AGACCAAGAATTGGTTTTTTACCTTC-3’). PCR was initiated at 94°C for 10 min followed by 40 cycles of 60 sec at 94°C, 60 sec at 50°C, and 90 sec at 72°C.

### *In Vivo* Evaluation of the Symbiotic Pair of the Intestinal Pair for Ammonia-Removal Efficacy

To evaluate the ammonia-removal efficacy of selected intestinal microbes *in vivo*, NH_4_Cl was *i.v.* injected on the last day into the mice fed the symbiotic pair for 2 weeks at the dosage of 100 mg/kg of body weight to elevate blood ammonia (n=3 per group). The blood and fecal samples were collected at 1 h after injection of NH_4_Cl. The blood was centrifuged, and serum was collected for ammonia quantitation. After the sacrifice of animals, the brain and fecal samples were collected and homogenized. After homogenization, the samples were centrifuged and the supernatant was used for ammonia quantitation with an ammonia assay kit (AA0100; Sigma-Aldrich).

### *In Vivo* Evaluation of the Symbiotic Pair of the Intestinal Pair for Therapeutic Efficacy in Acute Hyperammonemia Model

To evaluate the therapeutic efficacy of ammonia-removal microbes, mice were grouped and treated with normal saline for the control group, thioacetamide (TAA) for the acute hyperammonemia group, and TAA and symbiotic pair microbes for the treatment group (n=3 per group). Acute hyperammonemia was induced by intraperitoneally injected TAA at 250 mg/kg body weight on day 0 and day 1. For the symbiotic pair treatment group, each culture of *L. reuteri* JBD400 and *S. rubneri* JBD420, 1×10^9^ CFU in 200 µL PBS, were orally administered to the mice daily for 40 days before injection of TAA. In day 2, mice were sacrificed and blood, brain tissue, and feces were collected and analyzed for ammonia quantity, histology, and neuronal apoptosis. For histological examination of mouse tissues, the brain and liver tissues from each group were immediately fixed with 10% buffered neutral formalin. The tissues were embedded in paraffin wax, sliced into 6 µm sections, deparaffinized, and cleared. Hematoxylin and eosin (H&E) staining were used for the histopathologic examination.

For neuronal apoptosis assay, brain tissue from each group was sliced into 6 µm sections by using a cryotome (Thermo Scientific). Fluorescent-labelled Inhibitor of Caspases (FLICA™) assay kit was used to quantitate apoptosis. The FLICA reagent was easy to enter and irreversibly binds to activated caspase-9 in cells. The stock concentrate of FLICA Caspase-9 reagent (1:50 DMSO) was diluted with 200 µL PBS. The diluted FLICA was directly added to samples and incubated for approximately 20 min. Slices were washed 3 times with apoptosis wash buffer. Then slices were stained with DAPI according to the protocol. Data were analyzed using a fluorescence microscope (Nikon).

### Statistics

 Data were represented as means ± SD. Statistical analyses were performed using GraphPad Prism 8 (GraphPad Software, La Jolla, CA, USA). Comparisons were made using Student’s t test between two groups. Analyses across multiple groups were made using a one-way analysis of variance (ANOVA) with Dunnett’s multiple comparisons *post hoc* test. P values less than 0.05 were considered to be significant.

The experiment data that support the findings of this study are available in this article and its **Supplementary Information** files.

## Data Availability Statement

The original contributions presented in the study are included in the article/**Supplementary Material**. Further inquiries can be directed to the corresponding authors.

## Ethics Statement

All of the procedures performed with animals were in accordance with the established guidelines and were reviewed and approved by the Ethics Committee of the Institution’s Laboratory Animal Center.

## Author Contributions

S-TH and HK conceived and designed the experiments. JL, CZ, and SK performed the experiments. J-RR, SL, and HH provided technical and material support. All authors contributed to the article and approved the submitted version.

## Funding

This work was supported by the JINIS Inc. under grant JINIS BDRD 410; Korea Forestry Promotion Institute under grant 2017028A00-1819-BA01; and Korea Ministry of SMEs and start-ups under Grant Technology Development Program S2950439.

## Conflict of Interest

Authors H-JK and SK were employed by the company JINIS Inc. Author H-JK was also employed by SNJ Pharma Inc.

The remaining authors declare that the research was conducted in the absence of any commercial relationships that could be construed as a potential conflict of interest.

The authors declare that this study received funding from JINIS Inc under Grant JINIS BDRD 410; Korea Forestry Promotion Institute under Grant No. 2017028A00-1819-BA01; and Korea Ministry of SMEs and Start-ups under Grant Technology Development Program S2950439. The funder was not involved in the study design, collection, analysis, interpretation of data, the writing of this article, or the decision to submit it for publication.

## Publisher’s Note

All claims expressed in this article are solely those of the authors and do not necessarily represent those of their affiliated organizations, or those of the publisher, the editors and the reviewers. Any product that may be evaluated in this article, or claim that may be made by its manufacturer, is not guaranteed or endorsed by the publisher.

## Abbreviations

CNS, Central nervous system; GI, gastrointestinal; CFU, colony-forming unit; HA, hyperammonemia; TAA, thioacetamide.

## References

[B1] Adlimoghaddam A. Sabbir M. G. Albensi B. C. (2016). Ammonia as a Potential Neurotoxic Factor in Alzheimer's Disease. Front. Mol. Neurosci. 9, 57. doi: 10.3389/fnmol.2016.00057 27551259 PMC4976099

[B2] Butterworth R. F. (2020). “Hepatic Encephalopathy,” in The Liver: Biology and Pathobiology (NewYork, U.S: John Wiley & Sons Ltd.), 615–629. doi: 10.1002/9781119436812.ch48

[B3] Chaluvadi S. Hotchkiss A. T. Yam K. L. (2015). “Gut Microbiota: Impact of Probiotics, Prebiotics, Synbiotics, Pharmabiotics, and Postbiotics on Human Health,” in Probiotics, Prebiotics, and Synbiotics: Bioactive Foods in Health Promotion (Cambridge, U.S.A.: Academic Press), 515–523. doi: 10.1016/B978-0-12-802189-7.00036-8

[B4] Chen W. (2016). Generation Mechanisms of Hydrogen Cyanide and Ammonia in Human Exhaled Breath (Helsinki, Finland: Doctoral dissertation. Helsingin yliopisto: University of Helsinki). Available at: http://hdl.handle.net/10138/168771.

[B5] Chen X. D’Souza R. Hong S. T. (2013). The Role of Gut Microbiota in the Gut-Brain Axis: Current Challenges and Perspectives. Protein Cell 4, 403–414. doi: 10.1007/s13238-013-3017-x 23686721 PMC4875553

[B7] Chung H. J. Jae G. Y. Lee I. A. Liu M. J. Shen Y. F. Sharma S. P.. (2016). Intestinal Removal of Free Fatty Acids From Hosts by Lactobacilli for the Treatment of Obesity. FEBS Open Bio 6, 64–76. doi: 10.1002/2211-5463.12024 PMC4794792 27047743

[B6] Chung H. J. Nguyen T. T. Kim H. J. Hong S. T. (2018). Gut Microbiota as a Missing Link Between Nutrients and Traits of Human. Front. Microbiol. 9, 1510. doi: 10.3389/fmicb.2018.01510 30034384 PMC6043858

[B8] Diez-Fernandez C. Häberle J. (2017). Targeting CPS1 in the Treatment of Carbamoyl Phosphate Synthetase 1 (CPS1) Deficiency, A Urea Cycle Disorder. Expert Opin. Ther. Targets 21, 391–399. doi: 10.1080/14728222.2017.1294685 28281899

[B9] D’Souza R. Pandeya D. R. Hong S. T. (2012). Lactococcus Lactis: An Efficient Gram Positive Cell Factory for the Production and Secretion of Recombinant Protein. Biomed. Res. 23, 1–7.

[B500] Eckstein J. A. Ammerman G. M. Reveles J. M. Ackermann B. L. (2008). Analysis of Glutamine, Glutamate, Pyroglutamate, and GABA in Cerebrospinal Fluid Using Ion Pairing HPLC With Positive Electrospray LC/MS/MS. J. Neu. Meth. 171, 190–196. doi: 10.1016/j.jneumeth.2008.02.019 18433876

[B10] Estruch R. Ros E. Salas-Salvadó J. Covas M. I. Corella D. Arós F.. (2013). Primary Prevention of Cardiovascular Disease With a Mediterranean Diet. N. Engl. J. Med. 368, 1279–1290. doi: 10.1056/NEJMoa1200303 23432189

[B11] Ghallab A. Cellière G. Henkel S. G. Driesch D. Hoehme S. Hofmann U.. (2016). Model-Guided Identification of a Therapeutic Strategy to Reduce Hyperammonemia in Liver Diseases. J. Hepatol. 64, 860–871. doi: 10.1016/j.jhep.2015.11.018 26639393

[B12] Gruswitz F. Chaudhary S. Ho J. D. Schlessinger A. Pezeshki B. Ho C. M.. (2010). Function of Human Rh Based on Structure of RhCG at 2.1 Å. Proc. Natl. Acad. Sci. 107, 9638–9643. doi: 10.1073/pnas.1003587107 20457942 PMC2906887

[B13] Handlogten M. E. Hong S. P. Zhang L. Vander A. W. Steinbaum M. L. Campbell-Thompson M.. (2005). Expression of the Ammonia Transporter Proteins Rh B Glycoprotein and Rh C Glycoprotein in the Intestinal Tract. Am. J. Physiol. Gastrointest. Liver Physiol. 288, G1036–G1047. doi: 10.1073/10.1152/ajpgi.00418.2004 15576624

[B14] Huh L. Farrell K. (2011). “Urea Cycle Disorders,” in The Causes of Epilepsy: Common and Uncommon Causes in Adults and Children (Cambridge, United Kingdom: Cambridge University Press), 246.

[B15] Jin Y. Singh P. Chung H. J. Hong S. T. (2018). Blood Ammonia as a Possible Etiological Agent for Alzheimer’s Disease. Nutrients 10, 564. doi: 10.3390/nu10050564 PMC5986444 29734664

[B16] Jover-Cobos M. Khetan V. Jalan R. (2014). Treatment of Hyperammonemia in Liver Failure. Curr. Opin. Clin. Nutr. Metab. Care 17, 105–110. doi: 10.1097/MCO.0000000000000012 24281376

[B17] Kamei Y. Hatazawa Y. Uchitomi R. Yoshimura R. Miura S. (2020). Regulation of Skeletal Muscle Function by Amino Acids. Nutrients 12, 261. doi: 10.3390/nu12010261 PMC7019684 31963899

[B18] Karanfilian B. V. Cheung M. Dellatore P. Park T. Rustgi V. K. (2020). Laboratory Abnormalities of Hepatic Encephalopathy. Clin. Liver Dis. 24, 197–208. doi: 10.1016/j.cld.2020.01.011 32245527

[B19] Lee W. J. Hase K. (2014). Gut Microbiota–Generated Metabolites in Animal Health and Disease. Nat. Chem. Biol. 10, 416–424. doi: 10.1038/nchembio.1535 24838170

[B21] Lin R. Liu W. Piao M. Zhu H. (2017). A Review of the Relationship Between the Gut Microbiota and Amino Acid Metabolism. Amino Acids 49, 2083–2090. doi: 10.1007/s00726-017-2493-3 28932911

[B20] Li G. Z. Tio M. C. Pak L. M. Krier J. Seifter J. L. Tullius S. G.. (2019). Noncirrhotic Hyperammonemia After Deceased Donor Kidney Transplantation: A Case Report. Am. J. Transplant. 19, 3197–3201. doi: 10.1111/ajt.15545 31347272 PMC6864227

[B22] Liu J. Lkhagva E. Chung H. J. Kim H. J. Hong S. T. (2018). The Pharmabiotic Approach to Treat Hyperammonemia. Nutrients 10:140. doi: 10.3390/nu10020140 PMC5852716 29382084

[B23] Lourida I. Soni M. Thompson-Coon J. Purandare N. Lang I. A. Ukoumunne O. C.. (2013). Mediterranean Diet, Cognitive Function, and Dementia: A Systematic Review. Epidemiology 24, 479–489. doi: 10.1097/EDE.0b013e3182944410 23680940

[B24] Machado M. C. da Silva F. P. (2014). Hyperammonemia Due to Urea Cycle Disorders: A Potentially Fatal Condition in the Intensive Care Setting. J. Intensive Care 2, 1–5. doi: 10.1186/2052-0492-2-22 25908985 PMC4407289

[B25] Matsumoto S. Häberle J. Kido J. Mitsubuchi H. Endo F. Nakamura K. (2019). Urea Cycle Disorders—Update. J. Hum. Genet. 64, 833–847. doi: 10.1038/s10038-019-0614-4 31110235

[B26] Meng Z. Wang R. (2018). Production and Signaling Functions of Ammonia in Mammalian Cells. Gasotransmitters 12:101. doi: 10.1039/9781788013000-00101

[B27] Nagami G. T. Hamm L. L. (2017). Regulation of Acid-Base Balance in Chronic Kidney Disease. Adv. Chronic Kidney Dis. 24, 274–279. doi: 10.1053/j.ackd.2017.07.004 29031353

[B28] Nagpal R. Shively C. A. Appt S. A. Register T. C. Michalson K. T. Vitolins M. Z.. (2018). Gut Microbiome Composition in Non-Human Primates Consuming a Western or Mediterranean Diet. Front. Nutr. 5, 28. doi: 10.3389/fnut.2018.00028 29922651 PMC5996930

[B29] Nguyen T. T. B. Jin Y. Y. Chung H. J. Hong S. T. (2017). Pharmabiotics as an Emerging Medication for Metabolic Syndrome and Its Related Diseases. Molecules 22, 1795. doi: 10.3390/molecules22101795 PMC6151620 29064399

[B30] Nicaise C. Prozzi D. Viaene E. Moreno C. Gustot T. Quertinmont E.. (2008). Control of Acute, Chronic, and Constitutive Hyperammonemia by Wild-Type and Genetically Engineered Lactobacillus Plantarum in Rodents. Hepatology 48, 1184–1192. doi: 10.1002/hep.22445 18697211

[B31] Oja S. S. Saransaari P. Korpi E. R. (2017). Neurotoxicity of Ammonia. Neurochem. Res. 42, 713–720. doi: 10.1007/s11064-016-2014-x 27465396

[B32] Ott P. Vilstrup H. (2014). Cerebral Effects of Ammonia in Liver Disease: Current Hypotheses. Metab. Brain Dis. 29, 901–911. doi: 10.1007/s11011-014-9494-7 24488230

[B33] Pandeya D. R. Roshan D. Souza M. Rahman M. Akhter S. Kim H. J.. (2012). Host-Microbial Interaction in the Mammalian Intestine and Their Metabolic Role Inside. Biomed. Res. 23, 9–21. doi: 10.1172/JCI72335

[B34] Pourafshar N. Pourafshar S. Soleimani M. (2018). Urine Ammonium, Metabolic Acidosis and Progression of Chronic Kidney Disease. Nephron 138, 222–228. doi: 10.1159/000481892 29050011

[B35] Rahimi R. S. Rockey D. C. (2016). Hepatic Encephalopathy: Pharmacological Therapies Targeting Ammonia. Semin. Liver Dis. 36, 048–055. doi: 10.1055/s-0036-1571298 26870932

[B36] Saeedi B. J. Liu K. H. Owens J. A. Hunter-Chang S. Camacho M. C. Eboka R. U.. (2020). Gut-Resident Lactobacilli Activate Hepatic Nrf2 and Protect Against Oxidative Liver Injury. Cell Metab. 31, 956–968.e5. doi: 10.1016/j.cmet.2020.03.006 32213347 PMC7329068

[B37] Shen T. C. D. Albenberg L. Bittinger K. Chehoud C. Chen Y. Y. Judge C. A.. (2015). Engineering the Gut Microbiota to Treat Hyperammonemia. J. Clin. Investig. 125, 2841–2850. doi: 10.1172/JCI79214 26098218 PMC4563680

[B38] Singh P. Chung H. J. Lee I.-A. D’Souza R. Kim H. J. Hong S. T. (2018). Elucidation of the Anti-Hyperammonemic Mechanism of Lactobacillus Amylovorus JBD401 by Comparative Genomic Analysis. BMC Genomics 19, 1–14. doi: 10.1186/s12864-018-4672-3 29695242 PMC5918772

[B39] Sommer F. Bäckhed F. (2013). The Gut Microbiota—Masters of Host Development and Physiology. Nat. Rev. Microbiol. 11, 227–238. doi: 10.1038/nrmicro2974 23435359

[B40] Soria L. R. Brunetti-Pierri N. (2019). Ammonia and Autophagy: An Emerging Relationship With Implications for Disorders With Hyperammonemia. J. Inherit. Metab. Dis. 42, 1097–1104. doi: 10.1002/jimd.12061 30671986

[B41] Teperman L. W. Peyregne V. P. (2010). Considerations on the Impact of Hepatic Encephalopathy Treatments in the Pretransplant Setting. Transplantation 89, 771–778. doi: 10.1097/TP.0b013e3181d2fe66 20110852

[B42] van de Logt A.-E. Kluijtmans L. A. Huigen M. C. Janssen M. C. (2016). Hyperammonemia Due to Adult-Onset N-Acetylglutamate Synthase Deficiency. JIMD Rep. 31, 95–99. doi: 10.1007/8904_2016_565 27147233 PMC5272844

[B43] Weiner I. D. Mitch W. E. Sands J. M. (2015). Urea and Ammonia Metabolism and the Control of Renal Nitrogen Excretion. Clin. J. Am. Soc Nephrol. 10, 1444–1458. doi: 10.2215/CJN.10311013 25078422 PMC4527031

[B44] Wright G. Noiret L. Olde Damink S. W. Jalan R. (2011). Interorgan Ammonia Metabolism in Liver Failure: The Basis of Current and Future Therapies. Liver Int. 31, 163–175. doi: 10.1111/j.1478-3231.2010.02302.x 20673233

