# Supplementary material for: Treatment of Hyperammonemia by Transplanting a Symbiotic Pair of Intestinal Microbes
Source: Front Cell Infect Microbiol. 2022 Jan 5;11:696044. doi: 10.3389/fcimb.2021.696044 (PMC8766988; doi:10.3389/fcimb.2021.696044)
Supplement: Supplementary file 1 [file DataSheet_1.docx]

Supplementary Material

# Supplementary Tables

**Table S1.** The probiotic strains used for this study.

| Strain labeling | Strain name | Reference or source | |
| --- | --- | --- | --- |
| L1 | *Lactobacillus crispatus* JBL2-01301 | Gut Microbiota Bank | |
| L2 | *Lactobacillus gallinarum* JBL2-01901 | Gut Microbiota Bank | |
| L3 | *Lactobacillus acidophilus* JBD410 | Gut Microbiota Bank |  |
| L4* | *Lactobacillus amylovorus* JBD401 | Gut Microbiota Bank |  |
| L5 | *Lactobacillus intestinalis* JBL2-02401 | Gut Microbiota Bank |  |
| L6 | *Lactobacillus kalixensis* JBL2-02701 | Gut Microbiota Bank |  |
| L7 | *Lactobacillus jensenii* JBL2-02501 | Gut Microbiota Bank |  |
| L8 | *Lactobacillus delbruecki* JBL2-01401 | Gut Microbiota Bank |  |
| L9 | *Lactobacillus iners* JBL2-02301 | Gut Microbiota Bank |  |
| L10 | *Lactobacillus bulgaricus* JBL2-00901 | Gut Microbiota Bank |  |
| L11 | *Lactobacillus fermentum* JBD409 | Gut Microbiota Bank |  |
| L12* | *Lactobacillus reuteri* JBD400  (KACC Deposit No. 81122BP) | Gut Microbiota Bank |  |
| L13 | *Lactobacillus amylophilus* JBL2-00401 | Gut Microbiota Bank |  |
| L14 | *Lactobacillus farciminis* JBL2-01601 | Gut Microbiota Bank |  |
| L15 | *Lactobacillus alimentarius* JBL2-00301 | Gut Microbiota Bank |  |
| L16 | *Lactobacillus collinoides* JBL2-01101 | Gut Microbiota Bank |  |
| L17* | *Lactobacillus plantarum* JBD402  (KACC Deposit No. 81121BP) | Gut Microbiota Bank |  |
| L18 | *Lactobacillus parabuchneri* JBL2-03401 | Gut Microbiota Bank |  |
| L19 | *Lactobacillus kefiri* JBL2-02801 | Gut Microbiota Bank |  |
| L20 | *Lactobacillus buchneri* JBL2-00801 | Gut Microbiota Bank |  |
| L21 | *Lactobacillus lindneri* JBL2-02901 | Gut Microbiota Bank |  |
| L22 | *Lactobacillus sakei* JBL2-04001 | Gut Microbiota Bank |  |
| L23 | *Lactobacillus graminis* JBL2-02201 | Gut Microbiota Bank |  |
| L24 | *Lactobacillus coryniformis* JBD411 | Gut Microbiota Bank |  |
| L25 | *Lactobacillus pantheri* JBL2-03301 | Gut Microbiota Bank |  |
| L26* | *Lactobacillus rhamnosus* JBD406  (KACC Deposit No. 81123BP) | Gut Microbiota Bank |  |
| L27 | *Lactobacillus zeae* JBL2-04601 | Gut Microbiota Bank |  |
| L28 | *Lactobacillus casei* JBL2-01001 | Gut Microbiota Bank |  |
| L29 | *Lactobacillus brevis* JBL2-00701 | Gut Microbiota Bank |  |
| L30 | *Lactobacillus saerimneri* JBL2-03901 | Gut Microbiota Bank |  |
| L31 | *Lactobacillus salivarius* JBL2-04101 | Gut Microbiota Bank |  |
| L32 | *Lactobacillus ruminis* JBL2-03801 | Gut Microbiota Bank |  |
| L33 | *Lactobacillus sp* JBL2-04201 | Gut Microbiota Bank |  |
| L34* | *Lactococcus lactis* JBD404 | Gut Microbiota Bank |  |
| L35 | *Lactococcus garvieae* JBL3-00101 | Gut Microbiota Bank |  |
| L36 | *Lactobacillus casei* JBL2-01001 | Gut Microbiota Bank |  |
| L37 | *Lactobacillus parabuchneri* JBL2-03401 | Gut Microbiota Bank |  |
| L38 | *Lactobacillus gastricus* JBL2-02101 | Gut Microbiota Bank |  |
| L39 | *Lactobacillus ultunensis* JBL2-04401 | Gut Microbiota Bank |  |
| L40 | *Lactobacillus plantarum* JBD411 | Gut Microbiota Bank |  |
| L41 | *Lactobacillus johnsonii* JBP3-00401 | Gut Microbiota Bank |  |
| L42 | *Lactobacillus sharpeae* JBL2-05201 | Gut Microbiota Bank |  |
| L43 | *Lactobacillus antri* JBL2-00501 | Gut Microbiota Bank |  |
| L44 | *Lactobacillus oris* JBL2-03201 | Gut Microbiota Bank |  |
| L45 | *Lactobacillus agilis* JBL2-00201 | Gut Microbiota Bank |  |
| L46 | *Lactobacillus johnsonii* JBL2-02601 | Gut Microbiota Bank |  |
| L47 | *Lactobacillus gasseri* JBL2-02001 | Gut Microbiota Bank |  |
| L48 | *Lactobacillus bifermentans* JBL2-00601 | Gut Microbiota Bank |  |
| L49 | *Lactobacillus vaginalis* JBL2-04501 | Gut Microbiota Bank |  |
| L50 | *Lactobacillus mali* JBL2-03001 | Gut Microbiota Bank |  |
| S1 | *Streptococcus sobrinus* JBS15-02501 | Gut Microbiota Bank |  |
| S2* | *Streptococcus mutans* JBD423 | Gut Microbiota Bank |  |
| S3* | *Streptococcus ratti* JBD428 | Gut Microbiota Bank |  |
| S4* | *Streptococcus intermedius* JBD429 | Gut Microbiota Bank |  |
| S5 | *Streptococcus sanguis* JBD426 | Gut Microbiota Bank |  |
| S6 | *Streptococcus salivarius* JBS15-02201 | Gut Microbiota Bank |  |
| S7 | *Streptococcus sobrinus* JBS15-02501 | Gut Microbiota Bank |  |
| S8 | *Streptococcus peroris* JBS15-01701 | Gut Microbiota Bank |  |
| S9 | *Streptococcus cristatus* JBS15-00501 | Gut Microbiota Bank |  |
| S10* | *Streptococcus rubneri* JBD420  (KACC Deposit No. 81124BP) | Gut Microbiota Bank |  |
| S11 | *Streptococcus australis* JBD422 | Gut Microbiota Bank |  |
| S12 | *Streptococcus sanguinis* JBD425 | Gut Microbiota Bank |  |
| S13 | *Streptococcus constellatus* JBS15-00401 | Gut Microbiota Bank |  |
| S14 | *Streptococcus pasteuri* JBS15-01601 | Gut Microbiota Bank |  |
| S15* | *Streptococcus lutetiensis* JBD421 | Gut Microbiota Bank |  |
| S16 | *Streptococcus gallinaceus* JBD427 | Gut Microbiota Bank |  |
| S17 | *Streptococcus gordonii* JBS15-00901 | Gut Microbiota Bank |  |
| S18* | *Streptococcus pneumoniae* JBK1-00101 | Gut Microbiota Bank |  |
| S19 | *Streptococcus vestibularis* JBD424 | Gut Microbiota Bank |  |
| S20 | *Streptococcus pyogenes* JBS15-01901 | Gut Microbiota Bank |  |

*Indicate the strains for symbiotic pair study.

## Supplementary Figures

**
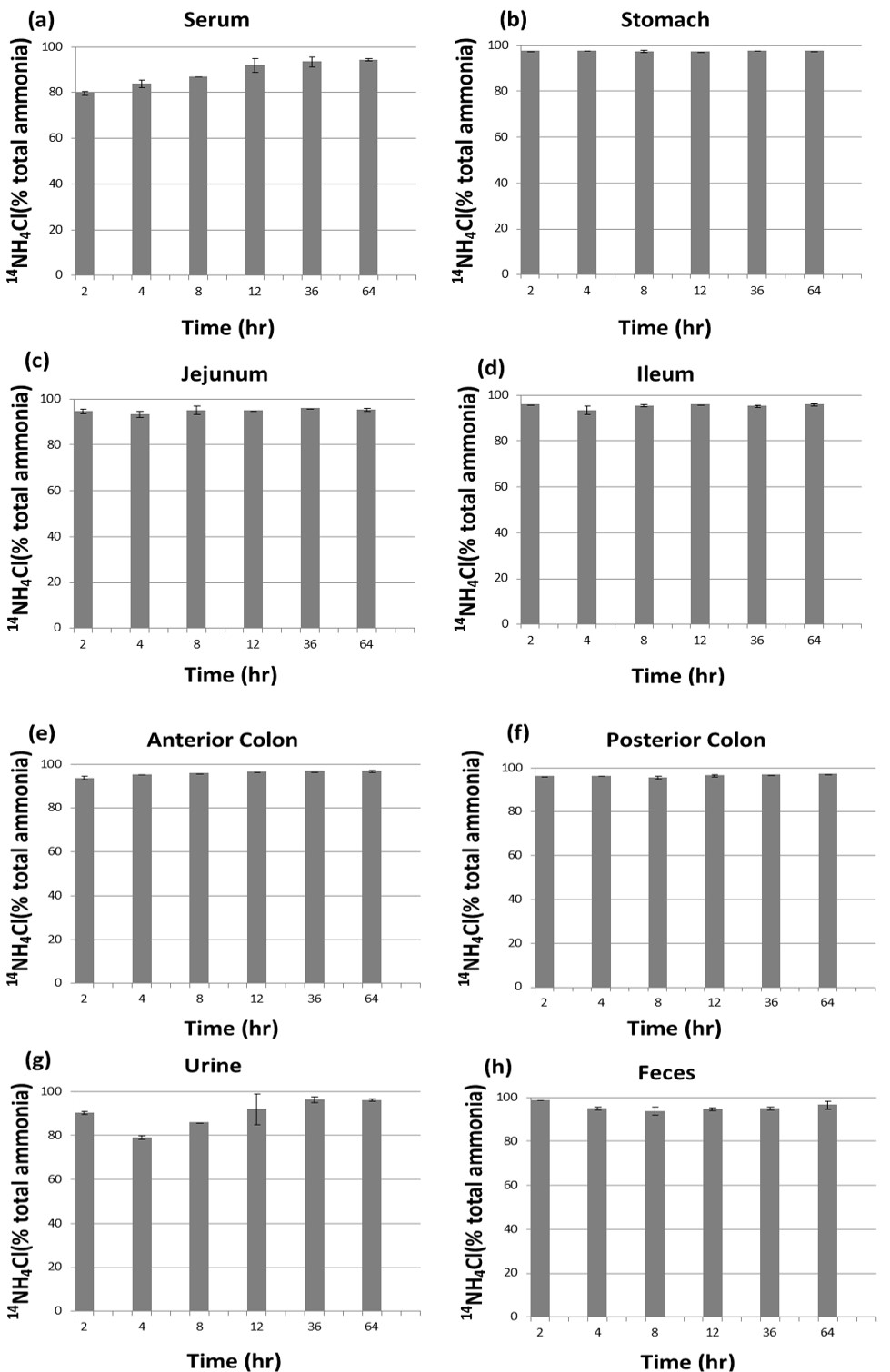
**

**Figure S1** **Distribution of intravenously injected ^14^NH_4_ in mouse organs**. After intravenous injection of ^15^NH_4_Cl into 4-weeks old C57BL/6 mice at a dose of 250 mg/kg of body weight, samples of blood, contents of the intestine (jejunum and ileum), contents of the colon (anterior colon and posterior colon), urine, and feces were collected at the indicated time. The concentration of ^14^NH was measured by analyzing with high-resolution LC-MS/MS in serum (a), stomach (b), jejunum (c), ileum (d), anterior colon (e), posterior colon (f), urine (g), and feces (h). All data were expressed as the mean ± SD, as indicated.

**
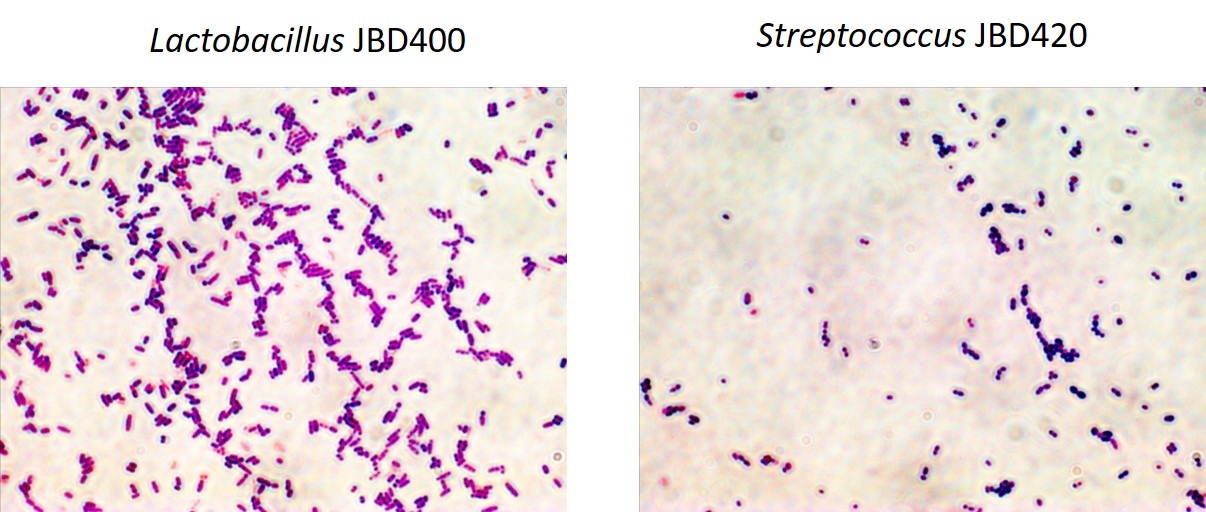
**

**Figure S2** **The Gram-staining result of *L. reuteri* JBD400 and *S. rubneri* JBD420.** The therapeutic efficacy of the symbiotic pair of *L. reuteri* JBD400 and *S. rubneri* JBD420 was further investigated for hyperammonemia.

**
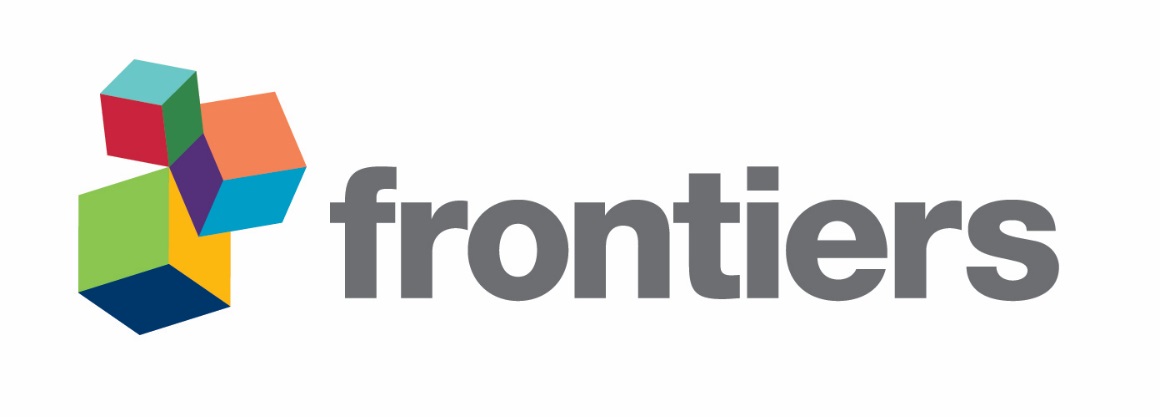
**
